# Primary health care team faultlines and team performance: the mediating role of knowledge sharing

**DOI:** 10.3389/fpsyg.2024.1362520

**Published:** 2024-06-20

**Authors:** Xinyu Bao, Yao Dai, Qian Wu, Wenjin Nie, Hongbing Tao

**Affiliations:** ^1^School of Medicine and Health Management of Tongji Medical College, Huazhong University of Science and Technology, Wuhan, China; ^2^Affiliated Jinhua Hospital, Zhejiang University School of Medicine, Jinhua, China; ^3^Shenzhen Health Development Research and Data Management Center, Shenzhen, China; ^4^The Affiliated Brain Hospital of Guangzhou Medical University, Guangzhou, China; ^5^Major Disciplinary Platform under Double First-Class Initiative for Liberal Arts, Research Center for High-Quality Development of Hospitals, Huazhong University of Science and Technology, Wuhan, China

**Keywords:** family doctor team, faultlines, knowledge sharing, team performance, primary health care

## Abstract

Family doctor teams, serving as health gatekeepers, are extensively advocated in China. Their composition, comprising a heterogeneous mix of professionals, contributes to a more comprehensive service, but also poses challenges. Consequently, scholarly interest has arisen in comprehending how these compositions, known as faultlines, influence team dynamics and outcomes. However, there is a lack of comprehensive exploration into how faultlines influence team members’ communication processes and knowledge sharing. This study aims to provide insights into the associations between faultlines in primary care teams and team performance, specifically exploring how knowledge sharing may mediate these effects, with the goal of revealing key insights to optimize contracted family doctor services. Survey data from 291 family doctor teams in China was utilized to test hypotheses, revealing a negative association between (social-category and information-based) faultlines and knowledge sharing. Team knowledge sharing acts as a mediator in the relationship between these faultlines and team performance. Our findings advance faultlines theory and emphasize the mediating role of knowledge sharing in elucidating the interplay between faultlines and team performance. These insights are crucial for fostering collaboration, managing faultlines, and enhancing healthcare team performance.

## 1 Introduction

Teams are commonly utilized in various industries as they are known to be highly efficient and creative. In healthcare, teams can utilize innovative solutions to address complex issues during the treatment process, which have the potential to enhance patient safety and efficiency (Paulus et al., 2008; Arana et al., 2017). Due to the shift in the direction of Chinese medical reform toward “strengthening the grassroots level,” the family doctor team, acting as the contracted service provider, now assumes the role of “gatekeeper” for resident health and is being widely promoted (Cai et al., 2022). Family doctor contracting services can deliver high-quality comprehensive care for the elderly, potentially enhancing the health-related quality of life for residents (Lai et al., 2021; Yuan et al., 2022). As the primary provider of family doctor contracting services, the family doctor team plays a pivotal role in ensuring the provision of high-quality care (Li et al., 2017). However, the family doctor contracting system is a recent policy in China, and how to improve the service quality and efficiency of primary health care teams is still being explored. Understanding contributing factors and mechanisms that affect outcomes in these teams has become a critical area of investigation (Huang et al., 2020).

A factor consistently associated with team dynamics and performance is the composition of the team (Mitchell et al., 2015). The family doctor team, as an interprofessional group, primarily consists of family physicians, nurses, and public health practitioners (assistants). Members of the family doctor team are obligated to provide both fundamental medical and public health services, encompassing personalized health guidance, outpatient appointments, and services specified in the contract (Wilkinson et al., 2020). In family doctor teams, family doctors primarily provide basic medical services, including the diagnosis and treatment of common and chronic diseases. Public health physicians focus on public health services, such as disease prevention, health education, and health management. Nurses provide both basic and personalized nursing services and assist family doctors and public health physicians in managing residents’ health (Yuan et al., 2019). This collaborative model seeks to expand the coverage and enhance the efficiency of medical services, while also ensuring continuous and personalized care (Yuan et al., 2022). This illustrates the benefits of interdisciplinary teams, though the potential implications of team diversity warrant further consideration. Although research into the impact of healthcare team configuration and collaboration on service quality has increased (Hysong et al., 2019; Mitchell and Boyle, 2021), studies focusing on these dynamics within family doctor teams in China are still limited.

As diversity research progresses from individual to subgroup levels, the concept of team faultlines, proposed by Lau and Murnighan (1998), hypothesizes that teams are divided into subgroups based on the alignment of members’ attributes or traits. These attributes or traits encompass not only demographic characteristics, such as gender and age, but also deeper aspects like values and attitudes. It is nearly impossible for these characteristics to align perfectly within a team. Consequently, team faultlines are prevalent in teams and are distinct from the broader concept of diversity. For example, consider two teams: the first team comprises two nurses aged 25 and two physicians aged 40, while the second team comprises one nurse and one physician aged 40, and one nurse and one physician aged 25. According to faultlines theory, the alignment of profession with age in the first group is more likely to disrupt team functioning compared to the second group, despite having the same diversity composition.

Previous research primarily classifies team faultlines into two types: social-category faultlines and information-based faultlines. The findings of previous studies frequently exhibit unclear patterns of effects (Jiang et al., 2012). The majority of scholars contend that social-category faultlines typically disrupt team function and performance, whereas information-based faultlines tend to yield a positive impact (Hutzschenreuter and Horstkotte, 2013; Cooper et al., 2014). Additionally, some argue that information-based faultlines can have detrimental effects (Bezrukova et al., 2012), while others propose that the connection between faultlines and outcomes is curvilinear (Rupert et al., 2016). With such an uncertain result, we proceeded to analyze the role of social-category (e.g., gender, age) and information-based (e.g., education, profession) faultlines in the family doctor team in accordance with the classification of previous studies (Bezrukova et al., 2010), aiming to gain a nuanced understanding of how they impact the processes and outcomes of primary healthcare teams. For measurement, scholars often employ objective indicators, using methods like Fau_g_ and ASW to calculate faultline strength (Ma et al., 2022; Chen et al., 2023; Sheffer Hilel et al., 2023). Additionally, other researchers use questionnaires to gauge team members’ perceptions of faultlines (Yao and Liu, 2023). Scholars generally agree that active faultlines perceived by members reflect underlying dormant faultlines (Qi et al., 2022). Moreover, it has been shown that dormant faultlines, quantified through objective attributes, similarly influence group outcomes as do active faultlines, and can strongly predict them (Zanutto et al., 2011; Thatcher and Patel, 2012). Consequently, this study employs dormant faultlines, quantified through objective metrics, as indicators of faultlines within family physician teams.

Scholars are actively exploring the role of team dynamics in studying the impact of the faultlines on team effectiveness, but the research still has limitations. The majority of the studies have focused on analyzing the role of team conflict and situational factors (Mitchell et al., 2019; Burmann and Semrau, 2022). However, team processes and cohesion are bolstered through communication behaviors (Bonito and Sanders, 2011). In the family doctor contract services, in addition to the coordination of the work content, the more important thing among communication between team members is the knowledge sharing. When a multidisciplinary group does its work, its performance depends not only on who knows what, but also on whether that knowledge is shared (Phillips et al., 2004). The strength of faultlines impacts the level of information discussion among team members (Meyer et al., 2011; Jiang et al., 2012). Further research is necessary to investigate team communication processes within the context of faultlines (Meyer et al., 2014).

**Figure 1:**
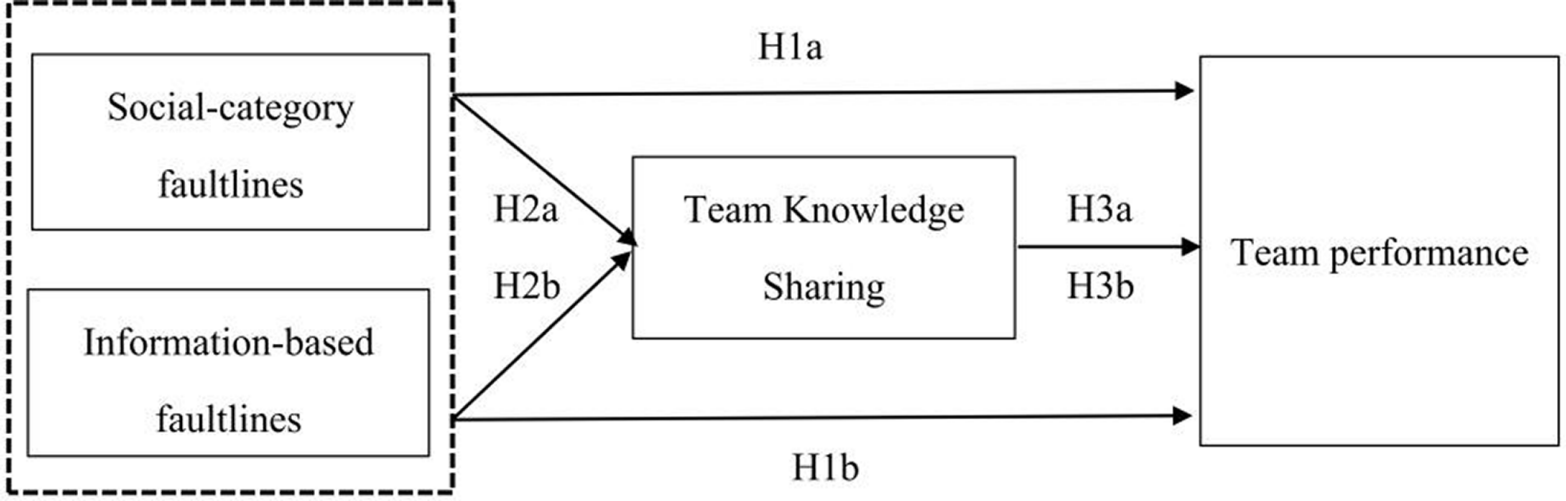
Conceptual framework.

Previous studies have highlighted the impact of faultlines on intrateam interactions (Ma et al., 2022; Szabo et al., 2024), but fewer studies have examined the influence of faultlines on multidisciplinary team dynamics (Mitchell et al., 2019; Sheffer Hilel et al., 2023), particularly within family doctor teams in China, has been underexplored. Understanding how faultlines within primary health care teams can either hinder or enhance team effectiveness is crucial for improving the quality of contracted family doctor services. This study further investigates the relationship between faultlines in family doctor teams and team performance, with a specific emphasis on the role of communication processes within this dynamic. Building upon earlier research on knowledge sharing in teams (Lemieux-Charles and McGuire, 2006), we assert that team faultlines impede team effectiveness by inhibiting knowledge sharing. Whether these faultlines are social-category or information-based, they diminish motivation and incentives for information sharing among members from various subgroups, ultimately undermining team performance. We predict a mediated relationship wherein team faultlines reduce team performance by impacting knowledge sharing, as illustrated in Figure 1. This not only advances the application of faultline theory but also highlights the mediating role of knowledge sharing in clarifying the interaction between faultlines and team performance in family physician teams. The subsequent section elucidates the rationale for the suggested correlation between team faultlines, knowledge sharing, and team performance and develop our hypotheses.

## 2 Theoretical background and hypotheses development

### 2.1 Team faultlines and team performance

#### 2.1.1 Team faultlines

Faultlines are described as “hypothetical dividing lines” that might divide a group into relatively homogeneous subgroups based on the demographic alignment of its members along one or more individual attributes (Lau and Murnighan, 1998). Rooted in social identity theory and self-categorization theory, faultlines prompt self and other categorizations into in-group or out-group members (Ashforth and Mael, 1989). Members tend to identify with in-group members while developing discrimination and prejudice against out-group members, which can influence group processes and outcomes. Strong faultlines tend to emerge at moderate team diversity levels, as homogeneous subgroups are improbable in either completely homogeneous or fully diverse teams (Lau and Murnighan, 1998). Teams with strong faultlines are more prone to team conflict (Jehn and Bezrukova, 2010), while strong faultlines can also have a negative impact on team performance, team satisfaction, and team creativity (Lau and Murnighan, 2005; Homan et al., 2008; Yao and Liu, 2023).

Depending on the hypothetical context and research emphasis, subgroups can be conceptualized based on the homogeneity of their members in identities, resources, or knowledge (Carton and Cummings, 2012). Attributes such as age, educational background, gender, and tenure are among the frequently employed variables in faultline studies (Thatcher and Patel, 2012). These attributes are combined to form social-category faultlines (e.g., age, gender) and information-based faultlines (e.g., educational background, tenure) which are mainly used by researchers (Bezrukova et al., 2009; Zimmermann, 2011; Ma et al., 2022; Qi et al., 2022). Furthermore, certain researchers have explored faultlines arising from non-demographic factors like team resources (Yao and Liu, 2023), personality traits (Gratton et al., 2007), and work location (Polzer et al., 2006). There is no consensus among researchers about the role of different faultlines in team outcomes. While most studies have affirmed the negative impact of social-category faultlines (Qi et al., 2022), the relationship between information-based faultlines and team outcomes has shown variability, exhibiting positive (Cooper et al., 2014), negative (Jiang et al., 2012), and no linear (Rupert et al., 2016). The effect of faultlines on team outcomes is also influenced by team type and attribute combination. Therefore, further research is necessary to comprehend the impact of different faultlines in different group.

#### 2.1.2 Social-category faultlines and team performance

According to the social identity theory, differences among team members, particularly identity-related difference (e.g., age, gender, race), heighten the probability of team members perceiving each other as part of distinct social categories (Meyer et al., 2011), thereby affecting individual perceptions of the boundaries of who is and who is not the part of the subgroup. Supporting or identifying with member out-subgroup might diminish the distinctiveness of their own subgroup, which then hinders the self-enhancement of subgroup members (Schölmerich et al., 2017). Thus, owing to the necessity for subgroup uniqueness or differentiation, subgroup members prefer to prioritize the goals of their own subgroup. As a result, individuals tend to trust and support members of their own subgroup more, displaying reduced concern for the objectives of other subgroups or the whole team (Wit and Kerr, 2002). Discrimination against out-subgroup is exacerbated when an “us vs. them” mentality arises, which fosters negative attitudes and hampers interaction between subgroups, perpetuating a vicious cycle that ultimately has a negative impact on team performance (Van Knippenberg et al., 2011). Bezrukova et al. (2016) also argue that, lack of convergence of social identities forms faultlines, where friction in interactions between members of different subgroups consumes time and resources that could have been used to achieve group goals and undermines group performance. Based on the preceding discussion, we propose the following hypothesis:

*H*1a: Social-category faultlines will be negatively associated with team performance.

#### 2.1.3 Information-based faultlines and team performance

Information-based faultlines exist within teams when there are alignments of task-related attributes (e.g., education level, functional background) (Cooper et al., 2014). These attributes suggest differences in work experience that may provide unique task-related insights and perspectives (Bezrukova et al., 2009). Following previous researchers who have stressed the important implications of information-based faultlines on performance in different work groups (Molleman, 2005; Bezrukova et al., 2012), we believe that strong information-based faultlines can negatively impact team cohesion, communication, and other team interaction processes, ultimately affecting team effectiveness. Groups with faultlines, as proposed by social identity and categorization theories, tend to divide into subgroups (Lau and Murnighan, 1998), potentially leading to conflicts between subgroups, impaired learning behavior, and disintegration of behavior (Li and Hambrick, 2005). Team members need to share information and expertise to achieve common goals. However, when subgroups form, it becomes more difficult for members to accept information and opinions from members of other subgroups (Lyu et al., 2022), and additional time must be spent reconciling divisions and conflicts, leading to further impairment of team efficiency (Yao et al., 2021). We thus predict that:

*H*1b: Information-based faultlines will be negatively associated with team performance.

### 2.2 Team faultlines and knowledge sharing

#### 2.2.1 Knowledge sharing

Knowledge is defined as the information, skills, and comprehension abilities acquired by individuals through education or experience. Knowledge sharing is the process of team members voluntarily sharing their acquired or created knowledge and expertise with others through direct communication or utilization of knowledge archive (Yu et al., 2013). Previous research has revealed some of individual factors that influence knowledge sharing. Obrenovic et al. (2020) suggests that altruism positively influences tacit knowledge sharing. Individuals with altruistic tendencies, deriving satisfaction from assisting others, are more inclined to share their knowledge. Optimistic members believe that organization has ability to achieve its goals. They prioritize knowledge sharing over possible negative effects resulting from collaborative or communicative efforts (Peterson, 2000). When members have psychological ownership of the given organization, they are more likely to engage in knowledge sharing and facilitate interactions among members (Zhang et al., 2022).

For organizations, knowledge sharing behaviors can have various positive impacts, such as increased performance and enhanced brand reputation (Lin et al., 2020; Zhang et al., 2021). However, previous research has indicated that knowledge sharing is an individual willingness that cannot be forced but can be encouraged, and it also come with participant costs. It is often easier to make knowledge sharing decisions when members have the belief that the expected benefits outweigh the cost (Bock et al., 2005). Family doctor teams require knowledge sharing among members to accomplish work tasks. However, according to social categorization theory, the division of internal and external groups may hinder collaboration among subgroups within a diverse team (Meyer et al., 2014). In this manner, diversity may impede the generation of task-relevant information and knowledge among team members, which can be detrimental to team performance (van Knippenberg et al., 2004). In other words, the existence of diversity, some members are classified as out-group members with whom he is unlikely to share knowledge even if there is a need for knowledge sharing.

#### 2.2.2 Social-category faultlines and team knowledge sharing

Bio-demographic characteristics, such as race and gender, are inherently present and typically unalterable (Milliken and Martins, 1996). This alignment may enhance the impact of any disparities and activate stereotypes (Lau and Murnighan, 1998). Team members, influenced by stereotypes arising from social-category faultlines, might segregate themselves and others into separate subgroups. Consequently, each subgroup becomes more inclined to provide support and heed other members within its own subgroup. Subsequently, these minor initial differences are amplified and exacerbated during the process of knowledge sharing, impeding team cooperation and coordination (Bezrukova et al., 2012). Social-category faultlines persist in heightening biases among individuals, prompting emotional responses such as distrust or antipathy, ultimately hinder knowledge sharing among members (van Knippenberg et al., 2004; Schotter et al., 2017). Lack of trust may lead individuals to be hesitant in sharing their knowledge and expertise (Currie and Kerrin, 2003). Thus, it is essential to anticipate potential social divisions among team members (Gratton et al., 2007). Therefore, we hypothesize that:

*H*2a: Social-category faultlines will be negatively associated with team knowledge sharing.

#### 2.2.3 Information-based faultlines and team knowledge sharing

Information-based faultlines may lead to negative consequences like prejudice (Lau and Murnighan, 1998). However, information-based faultlines also manifest and offer a way to address complex tasks. Team members can acknowledge the variances in knowledge stemming from diverse professional experiences or educational backgrounds, leveraging these differences when they respect and value diversity. At this point, members will tend to favor collaboration and integrating their unique resources, facilitating knowledge sharing (Rink and Ellemers, 2010). Generally, increased open and frequent communication tends to occur within teams adopting an egalitarian structure. When team with strong information-based faultlines, subgroups with more resources and information will have more power. Consequently, individuals with lesser power and status within the team are prone to silence and submission toward those possessing superior resources (Yao and Liu, 2023). Members tend to align with proposals endorsed by those in power, leading to decreased communication and discussion regarding alternative plans. Individuals perceiving higher authority often undervalue differing perspectives of others (Tost et al., 2013). The sharing of knowledge is optimal when it occurs within an egalitarian and inclusive context (Su et al., 2024). Consequently, this reduce the remaining team members’ motivation to share their thoughts and knowledge with others. Hence, teams exhibiting strong information-based faultlines are unlikely to foster free and open discussions. We therefore propose that:

*H*2b: Information-based faultlines will be negatively associated with team knowledge sharing.

### 2.3 The mediating role of team knowledge sharing

Based on the above hypotheses, we propose that knowledge sharing, serving as an activity facilitating the exchange of ideas and perspectives among team members, serves as a mediator for mitigating the adverse impacts of team faultlines on team performance. Information sharing within a team can enhance trust and cohesion, leading to improved socioemotional outcomes and ultimately enhancing team performance (Beal et al., 2003). Social-category faultlines evoked strong identification within subgroups but had a negative impact on the team’s overall sense of belonging (Polzer et al., 2006; Ma et al., 2022). The homogeneity of social attributes within subgroups creates communication barriers (Lu et al., 2018), which increases the gaps and prejudices across them, ultimately heightening the likelihood of relationship conflicts (Jehn and Bezrukova, 2010). Emotional and communication challenges stemming from social-category faultlines inhibit effective information sharing among team members. Additionally, significant disparities among classifications may provoke intense competition and hinder knowledge sharing (Zhu et al., 2019), ultimately limiting the capacity for team members to plan efficiently, coordinate activities, and solve problems swiftly (Jiang et al., 2012). Therefore, we propose the following hypothesis:

*H*3a: The relationship between social-category faultlines and team performance will be mediated by team knowledge sharing. Stronger social-category faultlines correspond to a reduction of team knowledge sharing, leading to poorer team performance.

Strong information-based faultlines increase intergroup bias within teams. Each subgroup may perceive the information shared by members of the outgroup as a challenge to the validity of their opinions (Yao et al., 2021). In particular, information from members with lower status within the team is often discounted (Gray et al., 2023). Thus, team members believe that expressing differing opinions in front of all members has more risks than benefits. Consequently, team members might opt for silence in the workplace to evade conflict. Rupert et al. (2016) also argues that misunderstandings between subgroup members, resulting from a lack of common ground, can significantly impair the efficacy of communication within a team. This hindrance can prevent the exchange of different perspectives, reducing the effective utilization of diverse viewpoints and compromising the team’s capacity to integrate unique expertise, essential for performance improvement (Gardner, 2012). Therefore, our study argues that those members with different perceptions tend to be viewed as outgroup members, potentially resulting in a tendency to withhold their knowledge, consequently leading to a detrimental impact on performance. Thus, we hypothesize the following:

*H*3b: The relationship between information-based faultlines and team performance will be mediated by team knowledge sharing. Stronger information-based faultlines correspond to reduced team knowledge sharing, leading to poorer team performance.

## 3 Methods

### 3.1 Procedure and sample

Our hypotheses were investigated using data collected from members of family doctor teams in three counties and cities in Hubei Province, China where family doctor contracting services are highly developed. A total of 32 township central health centers were selected with the help of local health administrative departments. The survey was distributed with the support of local health administration departments, with no compensation provided to the respondents. The paper questionnaires were collected. To ensure that participants were adequately informed and that their participation was entirely voluntary, the introductory section of the questionnaire described the purpose of the study, the scope of the information collection, potential privacy risks, and countermeasures. Participants were informed of their right to refuse the survey and that acceptance of the survey would be considered informed consent.

Our inclusion criteria were (a) the team consisted of more than three members; (b) all team basic information was provided by the team leader, such as the number of team members, team members’ names; and (c) members completed all questionnaires. Meanwhile, we made provisions on the validity of the responses to the questionnaire: the team leader of each team provided the basic team information, and more than half of the team members, at least four who answered all the questionnaire items. That is consistent with the previous research requirements (Harrison et al., 2002; Thatcher et al., 2003). The final sample comprised 1,319 individual team members from 291 teams, with an effective rate of 74.26%. In the final sample, team sizes varied from 4 to 9 individuals, averaging 5.55 members per team. Ages ranged from 22 to 78 years. The mean age was 43.64 years, with a standard deviation of 11.24 years. The specific breakdown was: 17.44% of participants were aged 22–30 years, 20.85% were aged 31–40 years, 37.22% were aged 41–50 years, 17.74% were aged 51–60 years, and 6.75% were aged 61 years and above. Of the participants, 569 (43.14%) were male and 750 (56.86%) were female. The average work tenure was 21.93 years (SD = 12.53), 38.74% of the team members had obtained junior college degree.

### 3.2 Measures

Our study variables were derived from established scales. Items originally in English were translated into Chinese using a back-translation procedure (Brislin, 1980). Given our hypotheses at the team level, we examined whether our data displayed team-level effects through analysis of variance and intraclass correlation coefficients. ICC(1) values indicate the proportion of between-group variance to total variance attributed to group membership, whereas ICC(2) values measure the reliability of average team perceptions (Klein and Kozlowski, 2000). We expected ICC(1) scores different from zero, with values close to 0.20 interpreted as high scores (Bliese, 2000). Glick (1985) proposed that ICC(2) values over 0.60 reflect high scores. Furthermore, an evaluation of interrater agreement (*r*_wg_) was conducted, with the goal of ensuring that all mean *r*_wg_ values surpass the acceptable 0.70 threshold necessary to validate aggregation (George, 1990).

#### 3.2.1 Team faultlines

To measure team social-category faultlines, we used two demographic characteristics: age and gender. And the information-based faultlines refer to three characteristics: educational level, profession, and work tenure. We selected the measurement for faultlines based on our theory and sample characteristics of small teams. Initially, the measurement was on the evaluation of faultlines strength. To assess faultlines strength, we employed the algorithm formulated by Thatcher et al. (2003) commonly utilized in faultlines research by various researchers (Lau and Murnighan, 2005; Bezrukova et al., 2009). For a group comprising n members, the potential subdivision into two subgroups can occur in S = (2^n-1^ − 1) ways. Each potential division will be computed to determine the ratio between the sum of squares among subgroups and the overall sum of squares. The analysis encompasses all conceivable splits where each subgroup encompasses at least two members; therefore, singular-member subgroups are excluded from consideration. The measure of faultlines represents the proportion of total variation in overall group attributes accounted for by the strongest group division (Molleman, 2005). The range of faultlines strength extends from 0 to 1, with higher values indicating stronger faultlines. Second, we measured faultlines distance (Bezrukova et al., 2009), delineating the degree of dissimilarity between subgroups calculated as the distance between subgroup centroids. Faultlines distance ranges from 0 to ∞, with a larger value indicating a larger distance.

The measurement of faultlines we used can be calculated using both continuous and categorical variables. We treated age and work tenure as continuous variables, while treating gender, educational level, and educational background as categorical variables. Following recommended procedure (Thatcher et al., 2003), we need to record categorical variables and rescale continuous variables, which allows categorical and continuous variables to be combined into a single distance measure that could then be used to measure faultlines. The categorical variables were converted to dummy variables and subsequently rescaled by multiplication with 1/ $\sqrt{2}$ , which can ensure that a difference in this categorical variable will contribute one to the overall distance between the two people. Continuous variables were rescaled by dividing all ages and work years by 10. A difference in gender or educational level or educational background as much to the distance between two people as a difference of 10 years in age or 10 years of work tenure.

Finally, following the previous research (Bezrukova et al., 2010), we standardized strength and distance scores by their respective maximum scores (Schaffer and Green, 1996), and performed a multiplication operation on these scores to explain the combined impact of faultlines strength and distance. This computation can significantly predict the active faultlines (Zanutto et al., 2011). The overall faultlines score was used in our analyses. Within our dataset, the social-category faultlinse scores ranged from 0.05 to 0.94, while the information-based faultlines scores spanned from 0.08 to 0.78.

#### 3.2.2 Team knowledge sharing

Five items of knowledge sharing designed by Bock et al. (2005) were used in our research, assessed through a 7-point Likert scale spanning from (1) “never” to (7) “quite frequently.” Two scale items were used to measure explicit knowledge, a sample item is “Members of the team regularly share work reports, official notification documents, and relevant institutional policies.” And three scale items were used to measure implicit knowledge, a sample item is “Members of the team frequently engage in the sharing of professional experience and expertise.” The intraclass correlation coefficients, ICC(1) and ICC(2), were found to be 0.35 and 0.71, respectively. Results obtained from the one-way ANOVA analysis (*F* = 3.464, *p* < 0.001) indicated significant differences in average team knowledge sharing. Additionally, the mean r_wg_ for this measure was 0.95, with one team having an r_wg_ of 0.63, which we retained. The team level Cronbach’s alpha (using item means) was 0.986.

#### 3.2.3 Team performance

We used three-item scale to measure team performance (Schaubroeck et al., 2007). Participants were asked “This team is very competent,” “This team gets its work done very effectively,” “This team has performed its job well.” Team performance was measured on a 7-point Likert scale anchored by 1 = strongly disagree and 7 = strongly agree. The ICC(1) was 0.25, and ICC(2) was 0.60. The outcomes from the one-way ANOVA analysis (*F* = 2.481, *p* < 0.001) suggested a satisfactory differentiation among teams. The mean r_wg_ for this measure was 0.93, with all teams exceeding the threshold of 0.70. In this study, the Cronbach’s alpha value for the scale was 0.971.

#### 3.2.4 Control variables

Team size and team tenure were included as control variables. Previous research has demonstrated the significance of team size in influencing team processes and outcomes (Gonzalez-Mulé et al., 2020), and larger teams are more likely to exhibit heterogeneity and to form subgroups (Shaw, 2004). Team size was reported by the leader of each family doctor teams. Team tenure was also included as a controlled variable because of its potential influence on team performance (Smith et al., 1994). More developed team are more likely to have a clear division of responsibilities among members, which in turn leads to superior performance compared to newer teams (Chong, 2007). Meanwhile, team tenure is widely used as a control variable in studies related to team faultlines and team performance (Mitchell et al., 2019; Sheffer Hilel et al., 2023; Yao and Liu, 2023), representing the average duration of members’ employment within their current team. Finally, in line with previous research on faultlines (Schölmerich et al., 2016), we also controlled for diversity effects, including diversity in age, gender, education, profession, and work tenure. Blau’s heterogeneity index was used to measure group heterogeneity for categorical variables (e.g., gender, Blau, 1977), while the coefficient of variation was utilized to measure group diversity for continuous variables (e.g., age, Allison, 1978).

## 4 Results

Table 1 displays the means and standards deviations for each variable, and correlations among model variables. Prior to the hypothesis testing, we evaluated a measurement model containing all items of the team knowledge sharing and team performance scales. The two-factor measurement model (*X^2^* = 31.592, *df* = 16, RMSEA = 0.058, CFI = 0.996, GFI = 0.973) got a good model fit. The VIF test was conducted on all variables entering the model. The results revealed that the range of VIF values was between 1.055 and 5.162, remaining below the multicollinearity threshold of 10. Subsequently, we investigate the relationships among team faultlines, team knowledge sharing, and team performance outcomes through hierarchical regression analysis.

**Table 1:** Descriptive statistics and correlations.

	Variables	1	2	3	4	5	6	7	8	9	10	11
1	Team size	1.000										
2	Team tenure	−0.053	1.000									
3	Gender diversity	−0.056	0.002	1.000								
4	Age diversity	0.024	0.043	0.024	1.000							
5	Education diversity	−0.009	0.004	−0.143*	0.120*	1.000						
6	Profession diversity	0.072	−0.209**	0.156**	0.048	0.097	1.000					
7	Work tenure diversity	−0.035	0.007	−0.048	0.827**	0.101	0.092	1.000				
8	Social-category faultlines	0.187**	0.045	0.082	0.653**	0.083	0.074	0.412**	1.000			
9	Information-based faultlines	0.067	0.147*	−0.075	0.637**	0.077	−0.094	0.588**	0.630**	1.000		
10	Knowledge sharing	0.073	0.101	0.022	−0.085	−0.033	0.016	−0.093	−0.132*	−0.153**	1.000	
11	Team performance	−0.030	−0.032	0.074	−0.064	0.033	0.104	−0.063	−0.184**	−0.199**	0.768**	1.000
	Mean	5.546	5.718	0.379	0.243	0.497	0.488	0.572	0.293	0.289	5.456	5.557
	S.D.	0.871	1.731	0.145	0.096	0.147	0.143	0.249	0.155	0.123	0.720	0.599

*N* = 291, *p < 0.05; **p < 0.01.

The results of our hypothesis testing are summarized in Tables 2, 3. In Model 2 and Model 4 of Table 2, the regression coefficients of social-category faultlines on team knowledge sharing (*β* = −0.815, *p* < 0.05) and team performance (*β* = −1.023, *p* < 0.01) were significantly negative, after including the control variables, supporting our Hypothesis 1a and 2a. And as shown in Table 3 Model 2 and 5, subsequent with incorporating the control variables, information-based faultlines were associated with a significant decrease in team knowledge sharing (*β* = −1.099, *p* < 0.05) and team performance (*β* = −1.138, *p* < 0.01). These findings are consistent with our Hypotheses 1b and 2b, suggesting potential negative associations.

**Table 2:** Hierarchical liner modeling with social-category faultlines.

	Team knowledge sharing		Team performance		
	Model 1	Model 2	Model3	Model4	Model5
Intercept	4.929***	4.781***	5.700***	5.514***	2.438***
Control variables					
Team size	0.069	0.094	−0.018	0.014	−0.047
Team tenure	0.045	0.047	−0.011	−0.008	−0.039**
Gender diversity	0.146	0.212	0.303	0.387	0.250
Age diversity	−0.695	0.161	−0.401	0.672	0.569
Independent					
Social-category faultline		−0.815*		−1.023**	−0.498*
Mediator					
Knowledge sharing					0.643***
F	1.875	2.507*	0.818	2.941*	77.982***
R-squared	0.026	0.042	0.011	0.049	0.622
Adjust R-squared	0.012	0.025	−0.003	0.032	0.614
R-squared change	0.026	0.017*	0.011	0.038**	0.573***

*N* = 291 unstandardized coefficients are reported. *p < 0.05; **p < 0.01; ***p < 0.001.

**Table 3:** Hierarchical liner modeling with information-based faultlines.

	Team knowledge sharing		Team performance		
	Model 1	Model 2	Model3	Model4	Model5
Intercept	4.957***	4.995***	5.559***	5.598***	2.377***
Control variables					
Team size	0.060	0.076	−0.028	−0.012	−0.061*
Team tenure	0.048	0.057*	−0.004	0.006	−0.031*
Education diversity	−0.137	−0.110	0.122	0.151	0.221
Profession diversity	0.233	0.106	0.454	0.323	0.255
Work tenure diversity	−0.269	0.055	−0.185	0.151	0.115
Independent					
Information-based faultline		−1.099*		−1.138**	−0.429
Mediator					
Knowledge sharing					0.645***
F	1.599	2.417*	1.086	2.579*	67.020***
R-squared	0.027	0.049	0.019	0.052	0.624
Adjust R-squared	0.010	0.028	0.001	0.032	0.614
R-squared change	0.027	0.021*	0.019	0.033**	0.572***

*N* = 291 unstandardized coefficients are reported. *p < 0.05; **p < 0.01; ***p < 0.001.

Furthermore, our proposal suggests that team knowledge sharing will serve as a mediator in both the association between social-category faultlines and team performance and the connection between information-based faultlines and team performance (Hypothesis 3a and 3b). According to Model 5 in Table 2, after entering team knowledge, the regression coefficient of social-category faultlines on team performance was still significantly negative (*β* = −0.498, *p* < 0.05). However, there was a decrease in the regression coefficient in comparison to Model 4 in Table 2. Moreover, in line with the approach proposed by Preacher and Hayes (2008), bootstrapping analysis with 5,000 times replications was employed to assess the indirect effect using SPSS PROCESS template model 4. The findings indicated a significant indirect association between social-category faultlines and team performance through team knowledge sharing (−0.524, bootstrapped 95% CI = [−1.043, −0.066], excluding 0). Hence, Hypothesis 3a garnered support.

According to Model 5 in Table 3, after entering both social-category faultlines and team knowledge sharing in the regression analysis, only team knowledge sharing was significant (*β* = 0.645, *p* < 0.001). Then, we tested the indirect mediating effect with bootstrap method. The results indicated a substantial indirect association between information-based faultlines and team performance through knowledge sharing (−0.709, bootstrapped 95% CI = [−1.269, −0.141], excluding 0), supporting Hypothesis 3b.

**Figure 2:**
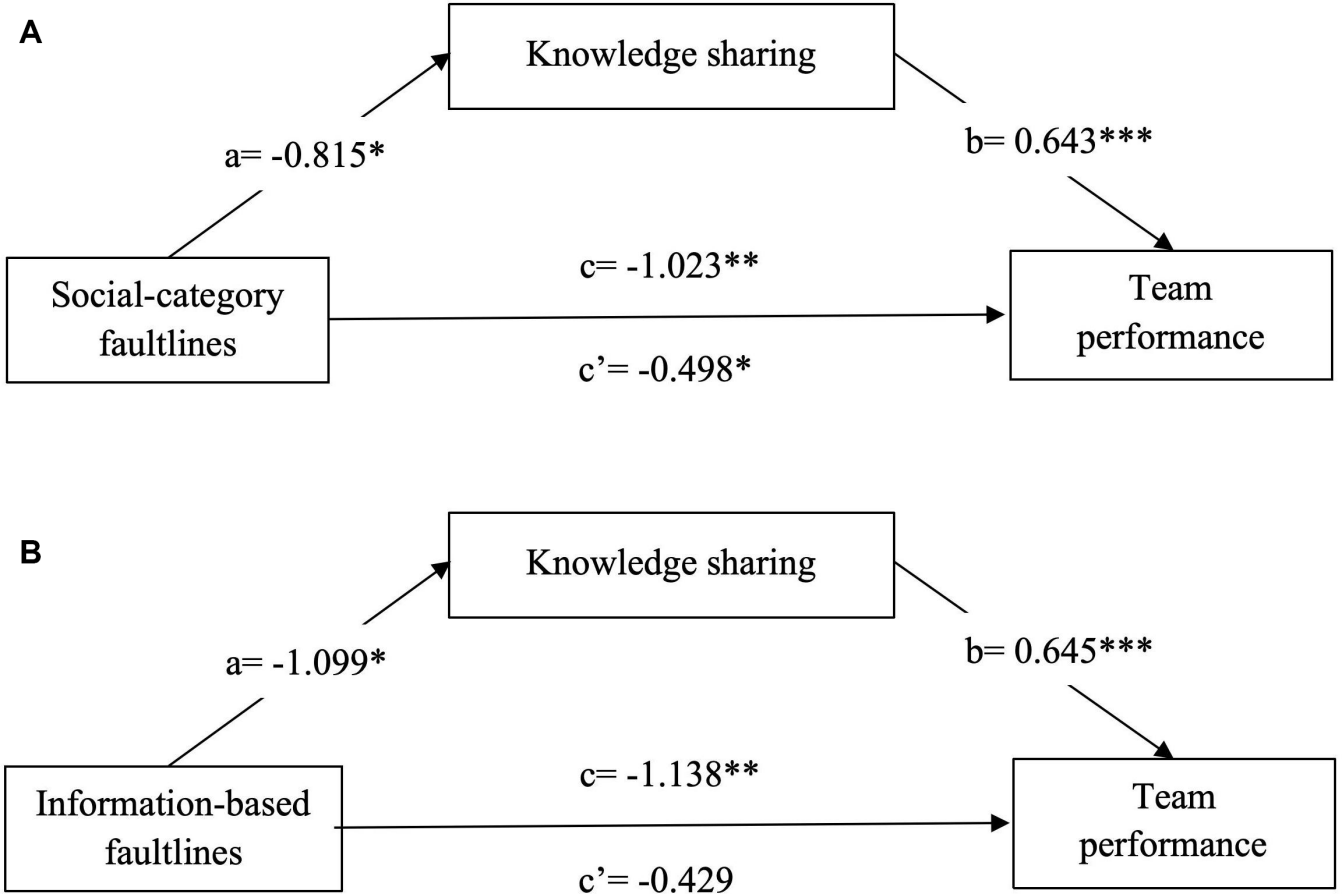
Mediation model **(A,B)** with knowledge sharing mediating the relationship between social-category/information-based faultlines and team performance (each path coefficient is symbolized by a, b, c, and c’); c represents the total effect of faultlines on team performance without the mediator in the model; c’ represents the direct effect of faultlines on team performance with the mediator in the model; each value provided is unstandardized. *p < 0.05; **p < 0.01; ***p < 0.001.

Further to simplify the model, the mediating effects of knowledge sharing in the relationship between social-category (Figure 2A) and information-based (Figure 2B) faultlines and team performance in a hierarchical linear regression model were shown in Figure 2.

## 5 Discussion

Our research applies faultline theory to explore the dynamics and performance of family doctor teams. This study investigates the associations between social-category and information-based faultlines with the processes and outcomes within family doctor teams, providing new insights into areas previously underexplored. The main findings of this study reveal that both social-category and information-based faultlines are significantly negatively associated with knowledge sharing and team performance. Additionally, the relationship between faultlines and team performance is found to be partially mediated by knowledge sharing. This fills a critical gap in understanding how faultlines function within the complex dynamics of family doctor teams.

### 5.1 Discussion of the results

Firstly, our study established that both social-category and information-based faultlines negatively correlate with team performance. The confirmed role of social-category faultlines in this study aligns with prior research. Previous scholarly efforts have strongly established that social-category faultlines are detrimental to team effectiveness (Thatcher and Patel, 2012; Burmann and Semrau, 2022). Social-category faultlines can create stereotyping and team conflict (Li and Hambrick, 2005; Thatcher et al., 2024), hindering smooth collaboration within family doctor teams, and negatively affecting team effectiveness. This study discovered that in family doctor teams, information-based faultlines have a negative effect on team performance. The direction of the role of information-based faultlines varies across previous researches (Cooper et al., 2014; Rupert et al., 2016; Georgakakis et al., 2017). In Chinese family doctor teams, the family doctor often assumes the role of team leader, thereby assuming responsibility for the management of the team’s activities (Yuan et al., 2019), which creates an invisible status difference within the team. When members are in the same subgroup as the family doctor, their knowledge and opinions may be more easily recognized, which may limit the diversity of perspectives needed for comprehensive problem-solving (Gray et al., 2023). This, in turn, may affect team performance.

Secondly, our study revealed a negative association between (social-category and information-based) faultlines and knowledge sharing within these teams, though previous research has not directly verified the relationship between faultlines and knowledge sharing. While previous research has not directly examined the relationship between faultlines and knowledge sharing, Ma et al. (2022) suggests that social-category faultlines tend to encourage knowledge hiding among team members, thereby creating an environment that is unfavorable for knowledge sharing. Information-based faultlines may result in members perceiving the risk of sharing information due to the discrepancy in status between subgroups (Anderson and Galinsky, 2006; Yao et al., 2021).

Finally, we found that team knowledge sharing acts as a mediator in the relationship between these faultlines and team performance. This observation aligns with the proposition put forth by Jackson et al. (2003), highlighting the critical role of knowledge sharing in mediating the effects of team diversity. This means that high levels of social-category or information-based faultlines are associated with reduced knowledge sharing among team members. Consequently, reduced knowledge sharing is linked to a decline in team performance.

In summary, our study not only advances the application of faultline theory but also emphasizes the significance of knowledge sharing as a mediator in elucidating the interplay between faultlines and team performance within family doctor teams. These insights offer valuable information for developing team management strategies designed to foster collaboration and mitigate the potential negative effects of faultlines, ultimately aiming to enhance the performance of healthcare teams.

### 5.2 Theoretical implications

Our study explores faultlines in family doctor teams, enriching the research on team faultlines in primary healthcare. It proposes a research framework that links social-category and information-based faultlines to knowledge sharing and team performance, revealing the mechanisms of family doctor team faultiness on team performance. This study further corroborates the negative association between social-category faultlines and team performance, aligning with the findings from previous studies (Burmann and Semrau, 2022; Zhang and Chen, 2023). Additionally, this study identified that the impact of information-based faultlines is also negative in family physician teams. Although it is not consistent with the results that most scholars believe that information-based faultlines have a positive impact on teams, it is supported by some research findings (Bezrukova et al., 2012; Han et al., 2023). This suggests that the impact of faultlines should be studied in the context of the specific type present within teams (Yao et al., 2021).

Our research also confirmed that both identity and information-based faultlines played roles in shaping team members interactions. Pronounced identity and information-based faultlines were negatively associated with team knowledge sharing. Our study verified the negative effect of social-category faultlines on team knowledge sharing, aligning with earlier research (Phillips et al., 2004). Information-based faultlines tend to highlight individuals’ social identities and might impede their interactions with outgroup members (Jiang et al., 2012). Our study, alongside previous research, substantiates the notion that faultlines arising from the convergence of social-category or information-based characteristics serve as better predictors of intragroup knowledge sharing. Importantly, our study contributes to the theoretical framework on faultlines by highlighting their association with the dynamics of team knowledge sharing and their potential effects on team performance. These insights contribute to advancing the theoretical understanding of faultlines.

### 5.3 Practice implications

The identified negative associations between faultlines and team knowledge sharing underscore the necessity of evaluating faultlines during team formation. Prior research also recommends that managers focus on team composition to minimize the likelihood of forming homogeneous subgroups (Burmann and Semrau, 2022). In our results, the social-category faultlines of the family doctor teams affected both team knowledge sharing and team performance. Hence, when building a team, managers should recognize the importance of team composition, and be more careful about matching attributes such as gender and age.

While real work environments tend to possess more intricate structures, the diverse attributes among potential team members may mean that it is impractical to form teams without structural faultlines (Jiang et al., 2012). In instances where faultlines are prevalent, managers could aim to mitigate their detrimental effects by establishing a strong sense of collective identity among team members (Bezrukova et al., 2009). To ensure comprehensive management of primary care and prevention (Liu et al., 2019), the family doctor team is a multidisciplinary team, so that the presence of team professional related faultlines was inevitable. To enhance interaction and effectiveness within the family doctor team, they can use other ways, such as the presence of a transformational and empowering leader or improving the team atmosphere, to alleviate the influence of the information-based faultlines on knowledge sharing (Stewart, 2006).

We also find the positive relationship between team knowledge sharing and team performance. Knowledge sharing appears to moderate the association between team faultlines and team performance. Creating an environment that promotes knowledge sharing within family doctor teams may enhance the team’s ability to utilize its collective expertise, potentially improving the quality of care provided to patients. Institutions can reduce communication barriers and facilitate sharing among team members through policy support and technology integration. For example, holding regular knowledge-sharing meetings and encouraging member participation, and using management systems to streamline internal information-sharing processes.

### 5.4 Limitation and further research

While the study results provide initial empirical evidence, certain limitations indicate the need for further research. Primarily, our inability to collect longitudinal data restricted our capacity to trace temporal patterns or establish causative connections. Longitudinal studies would provide a comprehensive understanding of how these variables evolve over time, allowing for a more nuanced understanding of their relationships. Thus, we encourage future study using a longitudinal design, which could make a meaningful contribution to clarifying the causal relationship between team faultlines, knowledge sharing, and team performance. Secondly, in this study, due to the unavailability of specific data on patient outcomes for the teams, we resorted to self-reported measures of team performance. This approach may have introduced reporting bias, potentially affecting the objectivity of our findings. Additionally, we observed that members’ self-assessments of team knowledge sharing and team performance were generally on the higher side, especially when averaged at the team level. This aggregation may lead to a range restriction in the variables, potentially affecting the results. Therefore, future research should explore additional ways to access data, such as obtaining patient outcome data directly from healthcare information systems, to enhance the quality and reliability of the research.

Future research can leverage the theoretical arguments and empirical substantiation presented in this study to enhance our comprehension of the consequences of faultlines. Our study focused on exploring the consequences of social-category and information-based faultlines. While our study primarily examined faultlines based on social-category and information-based divisions, which represent the most frequently researched division attributes. Exploring faultlines constituted by non-demographic attributes or combinations thereof could unveil additional insights. Investigating faultlines emerging from diverse facets like work styles, cognitive approaches, or cultural disparities could enrich our comprehension of team dynamics. Furthermore, despite their strong correlation with activated faultlines, dormant faultlines must be perceived by the team to affect the interaction process. Therefore, future studies could analyze dormant and activated faultlines together to gain a comprehensive understanding of the impact mechanisms of faultlines. In addition, environmental factors, such as the influence of leadership dynamics and team climate, may modulate the effects of faultlines. Exploring how these external factors interact with faultlines could provide a more complete understanding of their impact on team functioning.

## 6 Conclusion

Despite its limitations, this study broadens the existing literature by exploring the relationship between primary health care team faultlines and team performance. The empirical findings indicate that both social-category and information-based faultlines within family doctor teams are negatively associated with team performance, with knowledge sharing serving as a mediator in this relationship. This study advances our understanding of the mechanisms that underlie the association between team faultlines and team performance in Chinese family doctor teams.

## Data availability statement

The raw data supporting the conclusions of this article will be made available by the authors, without undue reservation.

## Ethics statement

Ethical approval was not required for the studies involving humans because ethical review and approval were not required for the study on human participants in accordance with the local legislation and institutional requirements. The studies were conducted in accordance with the local legislation and institutional requirements. The participants provided their written informed consent to participate in this study.

## Author contributions

XB: Conceptualization, Data curation, Writing – original draft, Writing – review & editing. YD: Data curation, Formal analysis, Methodology, Writing – review & editing. QW: Writing – review & editing. WN: Writing – review & editing. HT: Conceptualization, Project administration, Resources, Supervision, Writing – review & editing.
